# Nuclear Lamins: A Molecular Bridge Coupling Extracellular Mechanical Cues to Intranuclear Signal Transduction and Gene Regulation

**DOI:** 10.3390/ijms27073258

**Published:** 2026-04-03

**Authors:** Shili Yang, Huaiquan Liu, Haiyang Kou, Lingyan Lai, Xinyan Zhang, Yunling Xu, Yu Sun, Bo Chen

**Affiliations:** College of Acupuncture and Massage, Guizhou University of Traditional Chinese Medicine, No.4, Dongqing Road, Huaxi District, Guiyang 550025, China; yangshili@gzy.edu.cn (S.Y.); liuhuaiquan@gzy.edu.cn (H.L.); koutom@163.com (H.K.); 13027814403@163.com (L.L.); zhangxy1215999@163.com (X.Z.); 18380595307@163.com (Y.X.); gzyztxss@163.com (Y.S.)

**Keywords:** nuclear lamins, mechanosensation, mechanotransduction, chromatin remodeling, epigenetic regulation, cell fate determination

## Abstract

Nuclear lamins are the core molecular bridge linking the extracellular mechanical microenvironment to intranuclear gene regulation, and play a central regulatory role in cellular mechanosensation and mechanotransduction. Here, we systematically integrate the latest global research progress on nuclear lamins, delineating the cascade regulatory mechanism by which lamins mediate the transmission of mechanical signals across the nuclear envelope and the subsequent regulation of chromatin remodeling and epigenetic modification, with a focus on the molecular characteristics and functional specificity of distinct nuclear lamin subtypes and their interaction modes with the Linker of Nucleoskeleton and Cytoskeleton complex (LINC complex) and chromatin. Existing studies have established that nuclear lamins are mainly divided into three categories: A-type lamins (Lamin A/C), B-type lamins (Lamin B1, B2), and germ cell-specific subtypes. Among these, A-type lamins directly determine the mechanical stiffness of the nucleus and serve as the core mediators of intranuclear mechanical signal transduction. Each subtype of B-type nuclear lamins has a well-defined, non-redundant functional division: Lamin B1 and Lamin B2 indirectly maintain nuclear structural stability and regulate epigenetic status by anchoring facultative heterochromatin and constitutive heterochromatin, respectively. Notably, Lamin A/C distributed in the nucleoplasm also bears significant mechanical tension, which challenges the long-standing view that the mechanical functions of nuclear lamins are restricted to the nuclear envelope region. After mechanical force is transmitted across the nuclear envelope to nuclear lamins via the LINC complex, it can regulate the spatial conformation of chromatin and epigenetic modifications, thereby determining core cellular life activities including proliferation, differentiation, and migration. Dysregulation of this pathway is closely associated with a wide spectrum of human diseases, including cardiovascular diseases, progeria, muscular dystrophy, and neurodevelopmental disorders. Taken together, this review systematically delineates the hierarchical regulatory network of the “LINC complex–nuclear lamina–chromatin” axis, advances our understanding of the fundamental principles of cellular mechanobiology, and provides a theoretical framework for deciphering the pathological mechanisms and developing targeted therapeutic drugs for related diseases.

## 1. Research Background

**Figure 1:**
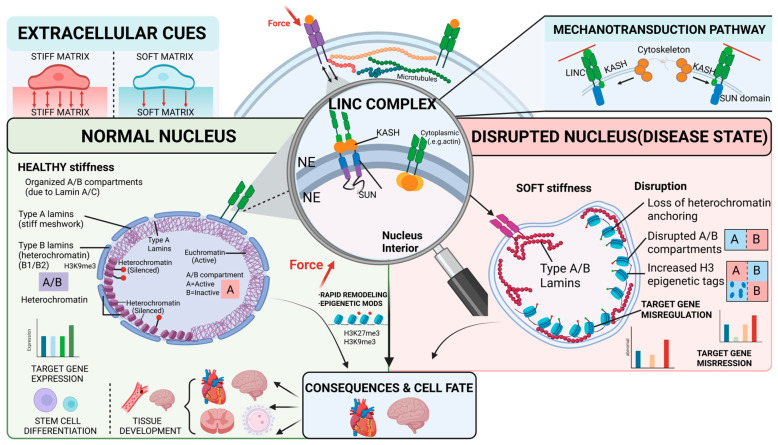
Schematic diagram of the core pathway of mechanosensation and signal transduction mediated by nuclear lamins.

Mechanosensation and mechanotransduction are core research topics in the field of cell mechanics. Mechanical forces sensed by cells are mainly derived from multiple biophysical cues, including the stiffness of the extracellular matrix (ECM)—a core intrinsic property of the cellular microenvironment—cell–cell interactions, and fluid shear stress. Importantly, matrix stiffness is not a mechanical force per se, but a core microenvironmental feature that regulates the ability of cells to sense and respond to mechanical signals [1]. Once extracellular mechanical signals are transduced into the cell via cell surface receptors such as integrins, they can modulate the epigenetic landscape through downstream signaling cascades, thereby dictating cell fate decisions [2]. This process of mechanotransduction plays a critical role in both physiological and pathological processes [3]. The nucleus is the core functional organelle of eukaryotic cells. It is not only the repository of genetic material and the regulatory center of gene expression, but also a key node that mediates intracellular mechanical signal transduction and modulates downstream biological effects. By integrating mechanical signals, the nucleus regulates core cellular processes including proliferation, differentiation and migration. Nuclear lamins are the core components of the filamentous protein network underlying the nuclear envelope. They not only play a pivotal role in maintaining the mechanical homeostasis of the nucleus, but also mediate the transmission of mechanical signals from the extracellular space into the nucleus. These proteins build the structural scaffold of the nucleus, and also participate in the regulation of multiple signal transduction pathways in the nucleus, thus becoming the key molecular bridges connecting the extracellular mechanical microenvironment and the regulation of intranuclear gene expression [4]. In recent years, with the development of Fluorescence Resonance Energy Transfer (FRET) technology, related studies have directly revealed the mechanical response characteristics of the nuclear lamina under dynamic mechanical strain at the protein level [5]. Ostlund et al. have quantitatively analyzed the molecular interaction between SUN proteins and Lamin A using acceptor photobleaching FRET technology, showing that the FRET efficiency of SUN1 and Lamin A (about 14–16%) is significantly higher than that of SUN2 and Lamin A (about 8–10%), and the difference between the two was statistically highly significant (*p* = 0.0017) [6]. This finding provides direct evidence for the specific interaction between the LINC complex and nuclear lamins, and reveals a tighter molecular association between SUN1 and Lamin A. Their specific binding may be an important molecular basis for the mechanical coupling between the LINC complex and nuclear lamins [6]. Further glutathione S-transferase (GST) pull-down and domain truncation experiments have confirmed that the binding site of SUN1 to Lamin A is located in amino acids 1–355 of its N-terminal nucleoplasmic domain, and the binding has strict subtype specificity—SUN1 only has a strong interaction with Lamin A, and has no significant binding to Lamin C, Lamin B1 and Lamin B2 [7]. The binding of Lamin A to SUN1 mainly depends on the amino acid sequence 607–646 of its C-terminal, and the C-terminal CaaX motif modified by farnesylation can significantly enhance the binding affinity of the two [8]. Other studies have shown that the dynamic mechanical response of the nuclear lamina is not regulated by a single factor, but by the synergistic regulation of the transnuclear envelope mechanical transmission efficiency of the LINC complex, the chromatin compaction state, and the anchoring mode of Lamina-associated domains (LADs) [9,10,11]. At the same time, Lamin A/C distributed in the nucleoplasm are not static structural components, and they also bear significant mechanical tension, which provides a key new theoretical perspective for systematically analyzing the mechanical response network of the nuclear lamina and the intranuclear “mechanochemical transduction” hub mechanism [5]. This review systematically explains the core mechanism of nuclear lamins as the molecular bridge of mechanosensation and signal transduction, and discusses the prospects of therapeutic strategies targeting this mechanical pathway. The relevant mechanism is shown in Figure 1.

## 2. Mechanisms of Nuclear Lamins in Mediating Mechanical Signal Sensation and Transduction

### 2.1. Molecular Structure and Physiological Functions of Nuclear Lamins

Nuclear lamins, the core structural components of the nuclear lamina in eukaryotic cells, belong to the type V intermediate filament protein family. They form a fibrous network structure under the inner nuclear envelope, which not only provides mechanical stability for the nucleus, but also deeply participates in physiological processes such as chromatin organization, gene regulation and signal transduction [12]. Their structure includes three functional domains: the head region responsible for inner nuclear envelope anchoring, the α-helical rod domain forming the structural skeleton, and the tail domain mediating chromatin attachment. These three functional domains together form the key hub for the transmission of mechanical force to the nucleus [13]. Among them, nuclear lamins can directly interact with chromatin through their tail domain. The conserved carboxyl terminal region of Lamin A/C can directly bind to DNA, and Isothermal Titration Calorimetry (ITC) experiments have shown that its dissociation constant is at the micromolar level, which provides a biochemical basis for the interaction between the nuclear lamina and chromatin [14]. Further studies have shown that the tail domain of mammalian Lamin C can directly bind to core histones, and the tail domain of Drosophila Lamin Dm0 can specifically recognize H2A-H2B dimers, suggesting that this interaction is highly conserved in evolution [15,16]. The binding of Lamin A to chromatin is strictly regulated by phosphorylation modification. Mitosis-specific phosphorylation mediated by *Cdc2* kinase can promote the depolymerization of the nuclear lamina and separate the nuclear lamina from chromatin [17]. The interaction between nuclear lamins and H2A depends on the conserved sequences of the two, and this specific binding is the molecular basis for the anchoring of chromosomes to the nuclear lamina [18]. Nuclear lamins mainly include A-type nuclear lamins, Lamin A, Lamin C and minor subtypes Aδ10, Aδ50, and C2, encoded by the *LMNA* gene, as well as B-type nuclear lamins, Lamin B1, Lamin B2 and Lamin B3, encoded by *LMNB1* and *LMNB2* genes [19,20,21]. Compared with the deletion of Lamin A/C or Lamin B1, Lamin B2 deletion exerts a milder effect on nuclear lamina structure. In terms of spatial distribution, type A and B-type nuclear lamins form intertwined dimer filament networks respectively. Among them, Lamin B1 forms a loose external network near the inner nuclear envelope, while Lamin A/C form a denser internal network in the nucleoplasmic region. In contrast, the filament network formed by Lamin B2 has larger gaps and irregular structure [22].

Lamin A/C mainly participates in maintaining the mechanical stability of the nucleus, nuclear structure homeostasis and nuclear positioning, and plays a key regulatory role in physiological activities such as high-order chromatin organization during mitosis, epigenetic regulation, nuclear pore complex assembly, gene transcription, nuclear envelope disassembly and reassembly, DNA replication, DNA damage response, cell cycle regulation, cell differentiation, and polarization during cell migration [23]. Studies [24] have shown that Lamin A can directly regulate chromatin organization, and its deletion leads to increased chromatin mobility and highly dispersed spatial distribution, thus increasing the risk of genetic material entanglement. Lamin C, as an alternative splicing product of the *LMNA* gene, has overlapping functions with Lamin A, but also has unique biological effects. One of the main differences between them is that Lamin C can be directly synthesized without post-translational processing, while Lamin A is synthesized in the form of a precursor (prelamin A) [25]. The C-terminal of prelamin A contains a CaaX motif, which drives its post-translational farnesylation, followed by proteolytic cleavage of the 15 C-terminal amino acids to generate mature Lamin A [26,27]. This difference in post-translational modification means that mature Lamin A is anchored to the nuclear envelope via its hydrophobic C-terminus, whereas Lamin C is predominantly localized in the nucleoplasm [23,25]. During cell division, Lamin A is phosphorylated and dissociates from the nuclear envelope, driving the disintegration of the nuclear envelope, and reassembles at the nuclear envelope after division [28]. After cell division, Lamin C is first dispersed in the nucleoplasm, and then gradually migrates to the nuclear envelope region, participating in nuclear structure reconstruction and chromatin spatial organization [25,29], suggesting that it may have a unique function in nuclear structure reconstruction. The α-helical rod domain of Lamin Aδ10 (deletion of exon 10 of the *LMNA* gene) is shortened, which leads to increased flexibility of the filament network formed by it, and may reduce the conduction efficiency of the nucleus to mechanical load [30]; the specific mechanism underlying this effect remains to be further elucidated. Lamin Aδ50 (deletion of the terminal 150 amino acids, retaining the farnesylation site) is anchored to the inner nuclear envelope due to the hydrophobicity of the C-terminal. The unfolded structure of its filaments has structural variability, which enables it to buffer local mechanical tension; however, excessive accumulation will disrupt the uniformity of the nuclear lamina network and weaken the overall mechanical stability of the nucleus [31,32,33,34]. The localization pattern of germ cell-specific Lamin C2 differs significantly from that of other nuclear envelope proteins such as Lamin B1 and Lamin B2—it does not form a continuous marginal distribution around the nucleus, but rather forms intermittent domains in the nuclear envelope and is enriched at telomere attachment sites. Chromosome telomeres are permanently bound to these Lamin C2 enrichment sites and move dynamically along the nuclear envelope [35,36].

All B-type nuclear lamins are synthesized as precursors, and need to go through a complex post-translational modification process of the C-terminal CaaX box (CaaX), including farnesylation, carboxymethylation and partial cleavage, to finally generate mature Lamin B1 and Lamin B2 [37]. This series of post-translational modifications is not only involved in the protein maturation process, but also directly regulates the subcellular localization and structural stability of nuclear lamins and their interaction with chromatin, which is a prerequisite for B-type nuclear lamins to exert their intranuclear physiological functions. Notably, there are conserved chromatin binding sites in the tail domain of nuclear lamins, which can directly interact with core histones, and the above post-translational modifications can significantly regulate this binding process [16]. Studies on mouse neurons show that the farnesylation modification of Lamin B1 plays an important role in the retention of intranuclear chromatin during neuronal migration. Model mice expressing Lamin B1 that cannot undergo farnesylation modification will have severe neurodevelopmental abnormalities. During neuronal migration, the nuclear lamina is separated from chromatin, and it is difficult to maintain the normal localization and retention of chromatin in the nucleus, while the loss of Lamin B2 farnesylation will not cause the above phenotype [38]. A variety of lamin-dependent signaling and structural mechanisms can interact to jointly regulate the chromatin dynamics process, and the disorder of this regulatory process is also an important molecular basis for the pathogenesis of laminopathies [39]. In Drosophila melanogaster, Lamin B mutants lacking the CaaX motif can cause the remodeling of nuclear structure, which further suggests that the CaaX motif has an important conserved role in maintaining the function of B-type nuclear lamins [40]. Lamin B1 is encoded by the long arm of chromosome 5. As one of the main components of the nuclear lamina, it forms a dense filamentous network with other nuclear lamins, provides mechanical support for the nuclear envelope to resist deformation or rupture, and participates in the anchoring of nuclear pore complexes, which is very important to maintain the integrity of nuclear structure and function [41,42]. B-type nuclear lamins form an overlapping network with A-type nuclear lamins. Unlike A-type lamins, which can extend into the nucleoplasm, B-type lamins are predominantly localized to the perinuclear region [43,44]. Structural analyses have shown that B-type nuclear lamins have larger grid side length and node connection density than type A, among which Lamin B1 has more edge connections per grid surface than Lamin B2 [45]. Studies [46] have shown that the anchoring of chromatin in the perinuclear region depends on the interaction with Lamin B1, and its deletion will lead to changes in the distribution and compression state of chromatin in the nucleus, and then affect the process of apoptosis and differentiation. In addition, the expression level of Lamin B1 is directly related to cell proliferation ability, and its down-regulation during aging is one of the key markers for cells to exit the proliferation state [47]. The main physiological functions of Lamin B2 include maintaining the structural integrity of the nuclear envelope and nucleolus, and participating in cell proliferation, senescence, DNA damage repair and gene expression regulation [21,48]. In normal mitosis, it maintains genome stability by ensuring the accuracy of chromosome segregation [49]. Studies have found that inhibition of A-type nuclear lamins and type B nuclear lamin receptors can interfere with the separation of chromatin A/B compartments, especially affecting the localization of chromatin regions, while specific inhibition of Lamin B1 or B2 has different effects on compartmentalization [50,51]. Given the similarity of the structural domains of B-type nuclear lamins, studies have pointed out that Lamin B1 preferentially anchors facultative heterochromatin (heterochromatin that changes dynamically with cell state), while Lamin B2 specifically binds to constitutive heterochromatin (stably silenced heterochromatin). The two regulate the separation of chromatin A/B compartments through partition, with clear functional specificity and no extensive redundancy [52], and only have partial overlap in basic functions such as nuclear envelope structure maintenance [53]. The above phenotypic characteristics of Lamin B1 and Lamin B2 with specific functions and partition regulation of chromatin compartments are not a simple functional differentiation phenomenon, and there is a clear molecular binding and epigenetic regulation mechanism behind it. The structural and functional partition characteristics of Lamin B1 and Lamin B2 in heterochromatin regions have been directly verified by genome-wide nicking enzyme epitope-targeted DNA sequencing (NEED-seq) technology [54]. Specifically, the binding regions of Lamin B1 are significantly enriched in facultative heterochromatin regions carrying H3K27me3 modification, while Lamin B2 stably binds to constitutive heterochromatin regions carrying H3K9me3 modification [54]. Molecular mechanism studies have further confirmed that there are essential differences in chromatin binding motifs and histone modification preferences between Lamin B1 and Lamin B2, which provide key experimental evidence for their non-redundant epigenetic regulatory functions [55]. Among them, Lamin B1 tends to bind to DNA sequences rich in guanine/cytosine (G/C), and is highly correlated with H3K27me3 modification; Lamin B2 prefers DNA regions rich in adenine/thymine (A/T), and shows specific binding characteristics with H3K9me3 modification [54,55]. Lamin B3 is produced by alternative splicing of the *LMNB2* gene. Its N-terminal and α-helical rod domains are replaced by a unique 84 amino acid sequence, and it is only expressed in the male germ cell line [56]. Studies [57] have shown that Lamin B3 can reduce the structural stiffness of the perinuclear region, provide a more flexible nuclear structure for germ cells, and thus promote the completion of specific nuclear recombination events during spermatogenesis. In summary, the mechanical functional characteristics of each subtype are summarized in Table 1.

### 2.2. Molecular Basis of Mechanosensation Mediated by Nuclear Lamins

The core of mechanosensation mediated by nuclear lamins lies in the complete molecular network of “receiving–transducing–effecting”. The LINC complex, as a bridge for the transnuclear envelope transmission of mechanical force, provides the structural basis for load reception of nuclear lamins; the inherent mechanosensitive properties of nuclear lamins determine the efficiency and scope of load transmission; and the specific molecular link realizes the accurate conversion of mechanical load into intranuclear signals.

#### 2.2.1. LINC Complex: The Molecular Bridge for Transnuclear Envelope Transmission of Mechanical Force to the Nuclear Skeleton

LINC complex proteins span the Inner Nuclear Membrane (INM) and the Outer Nuclear Membrane (ONM). They mediate the transnuclear envelope transmission of mechanical force through the interaction between SUN domain (SAD1/UNC84 domain containing protein, SUN) proteins localized in the INM and KASH domain (Klarsicht, ANC-1 and Syne homology, KASH) proteins localized in the ONM [58]. The LINC complex is not a rigid mechanical bridge, but a dynamic strain buffering system [59]. Experiments using microfluidic stretching combined with FRET tension sensor have shown that within the physiological stretching range of 5–20% of cells, the LINC complex can transmit mechanical tension to the nuclear lamina, and the significant mechanical force borne by the nuclear lamina depends on the functional LINC complex [5]. When stimulated by mechanical overload, SUN2 can trigger the conformational rearrangement of the LINC complex through the disulfide bond rearrangement mediated by its conserved cysteine (including the dynamic change in intermolecular disulfide bond formed with the KASH domain) to buffer the overload tension of the nuclear envelope. This process is an important regulatory mechanism for the LINC complex to achieve the stability of mechanical transmission [60,61]. At the same time, this mechanical stimulation can increase the phosphorylation level of Lamin A/C by 1.5–2-fold within a few hours, and realize the adaptive regulation of nuclear stiffness by regulating the phosphorylation state of Lamin A/C. This regulatory process is closely related to the response of cells to the mechanical microenvironment [62]. SUN proteins (especially SUN2) regulate trimer assembly/dissociation through disulfide bonds [60,61,63]. The spectrin repeat sequence of KASH proteins has elastic stretching ability. Together, they buffer mechanical load and prevent nuclear envelope rupture caused by local mechanical overload [64,65,66,67]. Their dynamic conformational changes are regulated by the phosphorylation of nuclear lamins and the compression state of chromatin [68,69]. Further studies have confirmed that post-translational modification is the core switch regulating the interaction between SUN and nuclear lamins. During mitosis, *Cdk1* and *Plk1* can phosphorylate the Ser48/Ser333 and Ser138 sites at the N-terminal of SUN1 respectively. This phosphorylation directly disrupts the binding between SUN1 and Lamin A/C, dissociating SUN1 from the nuclear lamina without affecting the integrity of the SUN-KASH complex, thereby coordinating nuclear envelope disassembly and the functional maintenance of the LINC complex [68]. The phosphorylation modification of SUN2 is mediated by *CK2* kinase, which can reversely affect the stability of nuclear envelope architecture and mechanical transduction efficiency by regulating its ubiquitination and degradation level [70]. In addition, the farnesylation modification of Lamin A can significantly enhance its binding affinity for SUN1. The abnormally accumulated farnesylated progerin (Lamin Aδ50) in progeria has a 14.8-fold higher binding affinity for SUN1 than that of mature Lamin A, which leads to the abnormal accumulation of SUN1 in the nuclear envelope and destroys the homeostasis of mechanical transduction [8]. Among them, six main isoforms of KASH domain proteins have been identified, including Nesprin-1, Nesprin-2, Nesprin-3, Nesprin-4, KASH5 and lymphoid restriction membrane protein (Jaw1/LRMP) [71]. Among them, Nesprin-1 comprises the giant isoform Nesprin-1G and smaller isoforms Nesprin-1α and Nesprin-1β; Nesprin-2 includes the giant isoform Nesprin-2G and smaller isoforms Nesprin-2α, Nesprin-2β, and Nesprin-2γ. They have high sequence homology and structural elasticity. Their N-termini contain an actin-binding domain (ABD) consisting of tandem calponin homology (CH) domains, which can bind F-actin with high affinity via the CH domain. At the same time, the spectrin repeat sequence of their cytoplasmic segment can further enhance the cross-linking ability with actin filaments, forming a multi-path actin-binding interface [65,72], and realizing the effective transmission of mechanical force between the cytoskeleton and the nucleus [73]. Studies have shown that the abnormal function of Nesprin-1 can induce myocardial diseases and cytoskeletal defects by changing the homeostasis of Lamin A/C [74], while Nesprin-2 directly binds to Lamin A/C both in vivo and in vitro, and its nuclear envelope localization and function can be damaged by Lamin A/C mutations, suggesting a close interaction between them and nuclear lamins. In addition, Nesprin-2 also participates in the mechanical regulation of perinuclear cytoskeleton, and mediates the interaction between actin filaments and microtubules at the nuclear envelope level [75]. Its specific regulatory mechanism is that Nesprin-2 (especially its giant subtype Nesprin-2G) can act as an F-actin bundling protein and a kinesin-1 activating adapter, directly link kinesin-1 to F-actin, promote the transport of actin filaments along microtubule tracks, and realize the active cross-linking and dynamic coordinated regulation of the two cytoskeletons [75]. In mammalian cells, Nesprin can cooperate with spectraplakin and microtubule plus-end tracking protein EB1 to endow muscle nuclei with elastic properties [64], which also indirectly suggests the conserved function of the Nesprin family in the connection between the nuclear envelope and cytoskeleton [72]. Nesprin-3 contains Nesprin-3α and Nesprin-3β subtypes. Among them, Nesprin-3α can bind to plectin, while Nesprin-3β cannot bind to plectin due to the deletion of the key N-terminal binding region [76,77]. As an outer nuclear envelope protein, Nesprin-3 directly binds to the PLAT domain of the cytoskeletal linker protein plectin through its N-terminal cytoplasmic region [77], and then bridges the nuclear skeleton and intermediate filament cytoskeleton such as vimentin [76,78,79]. It participates in the localization and migration of subcellular structures [80], and enhances the compressive mechanical stability of the nucleus through the intermediate filament network [81,82]. Nesprin-4 has a simple structure, containing only one spectrin repeat and a C-terminal KASH domain [72,83]. Its cytoplasmic segment can directly bind to the KIF5B heavy chain of the microtubule plus-end-directed motor protein kinesin-1 through the specific leucine zipper-related LEWD motif [83,84], and then mediate microtubule-dependent nuclear polarity localization and cell polarization [71]. As a meiosis-specific coiled-coil protein, KASH5 can activate the dynein adapter molecule, transmit microtubule mechanical force to meiotic chromosomes, and is very important for chromosome synapsis and reproductive function [85]. LRMP is localized in the endoplasmic reticulum and outer nuclear envelope, and regulates nuclear positioning through the dual interaction of SUN proteins and microtubules [86]. SUN domain proteins are divided into five family members. Only SUN1 and SUN2 are widely distributed in various cells, and SUN3, SUN4 and SUN5 are specifically expressed in sperm cells [87]. Both SUN1 and SUN2 have a nucleoplasmic N-terminal transmembrane region, a C-terminal conserved SUN domain and an upstream coiled-coil domain. They bind to KASH proteins through the two terminal domains to build a bridge between the nuclear cortex and the cytoskeleton. Among them, SUN1 mainly acts on the microtubule cytoskeleton and regulates microtubule dynamics and actomyosin contractility. Its deletion can lead to accelerated microtubule depolymerization and reduced mechanical force transmission efficiency [88]. Further studies have confirmed that SUN1 interacts specifically only with Lamin A, and does not bind to other Lamin subtypes or B-type nuclear lamins [7]. Structural and biochemical experiments further confirm that the tight binding of SUN1 to the nuclear lamina also depends on the core motif of amino acids 209–228, which is the key structural basis for SUN1 to be distinguished from SUN2 and realize stable anchoring of the nuclear envelope [89]. SUN2 mainly regulates actin homeostasis. Its down-regulation can lead to reduced F-actin polymerization, nuclear envelope blebbing and telomere dislocation, and reduce the transmembrane mechanical force transmission efficiency of the cytoskeleton [60,90]. Other studies show that the deletion of Lamin A/C will interfere with the nuclear positioning of SUN2, indicating that there is functional synergy between them [91,92]. Different from SUN1, the nuclear envelope localization of SUN2 mainly depends on the expression of Lamin A, and their interaction can be destroyed by some pathogenic mutations of Lamin A (such as EDMD-related R453W and H222P mutations), which is also an important molecular mechanism of abnormal mechanical transduction in laminopathy. In summary, the mechanical force conduction path can be summarized as follows: ECM stress is transmitted to the outer nuclear envelope KASH protein through the cytoskeleton, and then relayed to nuclear lamins and intranuclear chromatin through SUN proteins in the perinuclear space, and finally regulates gene expression, DNA repair or nuclear morphological remodeling. The LINC complex has specificity for the transmission of mechanical load: Nesprin-1/2 receives the tensile load from the cytoskeleton through the actin binding interface, Nesprin-4 mediates microtubule-dependent nuclear positioning through kinesin-1, and KASH5 specifically transmits the load related to chromosome synapsis during meiosis [71,85]. These specific transmission mechanisms respectively realize the differential sensing and integration of the contractile tension of actin filaments, the dynamic traction of microtubules, and the compressive support force of intermediate filaments by the LINC complex, ensuring that the mechanical signals received by nuclear lamins are accurately matched with the physiological state of cells, and laying a specific foundation for subsequent load transmission.

#### 2.2.2. Mechanosensitive Properties of Nuclear Lamins

Nuclear lamins provide the core mechanical stiffness for the nucleus, which is essential for the normal physiological activities of cells, and can effectively resist nuclear deformation and genome disturbance induced by mechanical force [93,94]. The nuclear lamina is the core structure that determines nuclear stiffness, among which Lamin A/C plays a core mechanical regulatory role. Many studies have confirmed through magnetic twisting cytometry and other technologies that the decrease in Lamin A/C expression is directly positively correlated with the decrease in nuclear stiffness [95]. Magnetic twisting cytometry can apply controllable stress to the cell surface through ligand-coated ferromagnetic microbeads. Its parameters have a good linear range, and can accurately quantify the mechanical properties and force transmission efficiency of cells and subcellular structures [96,97]. As the core structural components of the nuclear lamina, Lamin A and C directly determine nuclear stiffness. Their expression levels are linearly correlated with the nuclear bulk modulus. The nuclear bulk modulus can change in the range of 2–4 MPa with the expression level of Lamin A/C, and the higher the local Lamin A/C density at the nuclear envelope, the lower the deformation degree of the nucleus in the corresponding region [95]. At the same time, Lamin A/C regulates the efficiency of cytoplasm-to-nucleus mechanical transmission mediated by the LINC complex. Compared with wild-type cells, Lamin A/C knockout (*Lmna*^−/−^) cells have impaired force transmission function, and there is an obvious difference in standardized nuclear strain between the two [98]. Nesprin-3, as an outer nuclear envelope protein that specifically anchors the intermediate filament network in the LINC complex, achieves specific binding with intermediate filaments such as vimentin and desmin through the bridging protein plectin, forming the mechanical connection basis between the nucleus and intermediate filaments [76,78]. When the interaction between Nesprin-3 and the intermediate filament network is abnormal, it will significantly weaken the buffering capacity of the nucleus to compressive stress, leading to an increase in nuclear deformation rate [99,100]. This phenomenon further suggests that the cytoskeleton can affect the nuclear lamina and participate in the regulation of nuclear mechanical homeostasis through the mechanical transmission pathway formed by the LINC complex. The above results confirm that there is a close mechanical synergistic regulatory relationship between Lamin A/C and the LINC complex, and they jointly participate in the transnuclear transmission of cellular mechanical signals and the maintenance of nuclear mechanical homeostasis [101]. The deletion of Lamin A/C can lead to a significant increase of 30–50% in the degree of nuclear deformation [98], and indirectly interfere with the nuclear mechanotransduction process by affecting the movement of transcription factors [102]. Studies have shown that nuclei lacking Lamin A/C exhibit a higher deformation rate under stretching stimulation [92] and lower cell survival rate under stretching stimulation, indicating that Lamin A/C plays an irreplaceable role in maintaining the tensile resistance of the nuclear skeleton. In contrast, the regulation of nuclear stiffness by Lamin B1/B2 is “indirectly dependent on chromatin conformation”. The single deletion of Lamin B1 only slightly reduces nuclear stiffness (about 10–15%), but it will lead to chromatin decompaction by destroying the anchoring of facultative heterochromatin to the nuclear envelope, and indirectly weaken the buffering capacity of the nucleus to mechanical stress [11,46]. The combined deletion of Lamin B1/B2 will significantly reduce nuclear stiffness and impair the efficiency of mechanical force transmission [103], suggesting that the two have synergistic rather than redundant functions in the mechanical network [54], and their functions are more focused on maintaining the integrity of nuclear envelope structure rather than mechanical properties. Lamin B2 has similar functions to Lamin B1. They regulate nuclear structure by anchoring chromatin to the perinuclear region. The deletion of Lamin B1 will lead to the detachment of lamina-associated domains from the nuclear envelope, cause chromatin decompaction and increased chromatin mobility, destroy the spatial isolation of chromosome territories and A/B compartments, and then indirectly affect the response ability of the nucleus under physical stress [46]. In addition, nuclear lamins and intranuclear skeleton proteins form a “tug-of-war model”: Lamin B1 provides an outward anchoring force around the nucleus, and intranuclear skeleton proteins exert an inward pulling force. The two maintain the distribution and movement homeostasis of chromatin through mechanical balance, and jointly ensure the structural integrity of the nucleus under mechanical stimulation [46]. However, other studies have shown that Lamin B1 plays an important role in maintaining the integrity of nuclear structure and mediating the anchoring of the nucleus to the LINC protein complex [104,105], suggesting that it is potentially related to mechanical force transmission. Cruz et al. [106] found that the correlation analysis between the expression levels of single Lamin A, Lamin B1 and Lamin C proteins and the elastic/viscoelastic properties of cells showed that Lamin C protein had the strongest linear correlation with the cellular mechanical phenotype, while Lamin B1 and Lamin B2 had no significant effect on the mechanical properties at the nuclear and tissue levels. However, other scholars [11] have found that the deletion of Lamin B1 and Lamin B2 will also lead to changes in nuclear stiffness, and the loss of type A or B-type nuclear lamins will significantly impair the transmission efficiency of cellular mechanical signals, suggesting that B-type nuclear lamins may have both mechanical force-mediated function and mechanosensitive properties. In summary, Lamin A/C and B-type nuclear lamins have synergistically constructed the core framework of the nuclear mechanical response through direct and indirect pathways, laying a structural foundation for the intranuclear transmission and effect transformation of mechanical loads.

#### 2.2.3. Molecular Pathways for Mechanical Load Reception and Transmission by Nuclear Lamins

Mechanical force sensing, nuclear stiffness regulation, LINC complex dynamic assembly and epigenetic modification are not linear causal relationships, but rather form a multi-dimensional interaction network: nuclear stiffness provides a physical basis for mechanical force sensing, the dynamic conformational change in the LINC complex not only transmits force but also regulates nuclear stiffness, and epigenetic modification (such as histone acetylation) can reversely affect the assembly of nuclear lamins and the stability of the LINC complex, which ultimately jointly regulate the cellular mechanical response. The sensing and conduction of mechanical load by nuclear lamins are a multi-level collaborative process of “extracellular mechanical signal input–transnuclear envelope transmission–intranuclear signal conversion”. Through specific protein interaction, dynamic structural changes and two-way signal feedback, they realize the accurate conversion of mechanical force into biological effects. In this process, nuclear lamins have the dual functions of “mechanosensor” and “signal transduction hub”, forming the core pathway for the cellular mechanical microenvironment to regulate cell fate [5,107,108,109]. After the extracellular mechanical signal completes the transnuclear envelope transmission through the integrin–cytoskeleton–LINC complex pathway, nuclear lamins, as the core hub, realize the intranuclear transmission of mechanical load through multi-level molecular pathways, and rapidly translate physical signals into biological effects such as chromatin remodeling and gene expression regulation. SUN1 and SUN2 bind to nuclear lamins through their N-terminal nucleoplasmic domain. Among them, SUN1 specifically binds to Lamin A (not binding to Lamin B/C), while the nuclear positioning of SUN2 depends on Lamin A/C (higher dependence on Lamin A) [7,91]. These SUN proteins form the nuclear envelope–nuclear lamina–chromatin connection network together with Lamin A/C, Emerin, BAF and LAP2β. Among them, LAP2β is the key mediator for the transmission of mechanical force from the nuclear lamina to chromatin, and promotes gene expression by stretching chromatin regions, while BAF mainly stabilizes the nuclear structure and participates in mechanical force transmission by bridging DNA and nuclear lamins, and significantly enhances the stability and efficiency of load reception [110,111,112]. In addition, nuclear lamins can also form a synergistic interaction with nuclear envelope proteins such as Emerin [113], BAF and LAP2α to jointly build a complete mechanical force transmission network: K6-SUMOylation modification of BAF can enhance its binding to Lamin A/C and form a stable nuclear envelope–chromatin connection; LAP2α maintains the low-assembly and high-fluidity state of Lamin A/C in the nucleus, so that the nucleus can maintain the dynamic balance of flexibility and rigidity under stress. The above interactions further improve the intranuclear transmission path of mechanical load, and ensure the efficient transduction of mechanical signals and the homeostasis of nuclear structure and function [110,111]. Notably, the nanobody-based molecular tension FRET biosensor has directly confirmed that the nuclear lamina generally bears significant mechanical tension, and the tension depends on actomyosin contractility, LINC complex function and chromatin condensation state, and Lamin A/C distributed in the nucleoplasm also plays an important mechanical function [5,61].

Different subtypes of nuclear lamins have constructed specific intranuclear mechanical load transmission pathways through structural characteristics and functional division. Type A Lamin A/C directly transmits loads through the rigid dimer filament network formed by the α-helical rod domain, and its phosphorylation modification can dynamically regulate the transmission efficiency of mechanical signals by adjusting the polymerization state of dimers [13,28,95,98]. The heterodimer formed by Lamin C and Lamin A can further enhance the mechanical sensitivity of the filament network and play a synergistic role in load transmission [98,108,114]. Type B Lamin B1/B2 realizes the indirect transmission and distribution regulation of mechanical signals through chromatin anchoring—Lamin B1 can bind to the boundaries of chromatin Topologically Associating Domains (TADs), and mechanical force stimulation can induce preferential decompaction of TAD boundaries, thereby regulating the distribution range of intranuclear load [52,115,116]. Lamin B2 mediates the transmission of mechanical load to the intranuclear heterochromatin region through specific binding to constitutive heterochromatin [54]. The two work together to maintain the integrity of the higher-order structure of chromatin under mechanical force stimulation [46]. Germ cell-specific subtypes (Lamin C2/B3) reconstruct and transmit loads through unique structures. Lamin C2 (deletion of 112 amino acids at the N-terminal) transmits mechanical force during meiosis to chromosomes through dynamic binding with telomeres, and promotes homologous recombination [117]. Lamin B3 (splicing product of *LMNB2* gene, shortened α-helical rod domain) reduces the stiffness of the perinuclear region and provides mechanical flexibility for nuclear recombination during spermatogenesis [57,118]. After the mechanical load is transmitted through nuclear lamins, it is quickly converted into biological effects through chromatin remodeling and epigenetic regulation: at the level of chromatin remodeling, mechanical force can directly induce chromatin stretching within 15 s, and forces applied perpendicular to the long axis of the cell induce the greatest degree of chromatin stretching and the highest level of corresponding gene expression [119,120,121]. This rapid mechanical–biological conversion is based on the nuclear mechanical regulation network formed by the nuclear lamina and chromatin. They not only synergistically determine the overall mechanical response characteristics of the nucleus, but also accurately convert extracellular mechanical signals into intranuclear structural and functional changes through specific interactions and structural connections. Existing studies have confirmed that chromatin and Lamin A jointly determine two different mechanical response modes of the nucleus. Among them, chromatin dominates the mechanical response of the nucleus to small extension (<1 μm), and the degree of chromatin compaction and heterochromatin level can regulate nuclear stiffness, while the level of Lamin A/C controls the strain hardening characteristics of the nucleus during large extension [10]. The specific interaction between Lamin protein and chromatin has a clear molecular basis. Among them, the tail domain of Lamin A has a specific binding motif, which can directly bind to nucleosomes; the deletion of Lamin A/C will reduce the concentration of nucleosomes around the nucleus, and the interaction between Lamin and chromatin will directly affect chromatin structure remodeling and gene expression [55]. On the basis of this structure and mechanical regulation, the further transmission of mechanical load mediated by Lamin A/C will directly trigger the dynamic changes in chromatin compartment remodeling and epigenetic modification, and then realize the deep conversion of mechanical signals to transcriptional regulation. The load transmission mediated by Lamin A/C can induce the dissociation of LADs from the nuclear lamina to increase chromatin transcriptional accessibility [122]. At the level of epigenetic regulation, mechanical stress can induce the enrichment of Histone Deacetylase 3 (*HDAC3*) to perinuclear heterochromatin, leading to a decrease of about 60% in H4K16ac level [94], and promote the down-regulation of Lamin A/C expression to accumulate open chromatin epigenetic markers [123]. These effects ultimately regulate core physiological functions such as cell differentiation and apoptosis, while abnormal load transmission will lead to pathological states such as dilated cardiomyopathy and progeria [98,124,125].

## 3. Regulation of Chromatin Remodeling and Epigenetics by Nuclear Lamins

The regulation of chromatin remodeling and epigenetics by nuclear lamins is closely dependent on the synergy of their mechanical functions and structural distribution. Lamin A/C not only regulates epigenetics by anchoring chromatin [126,127], but also acts as a “mechanical damper” to buffer the response of chromatin to mechanical stress [98,128,129]. Its α-helical rod domains absorb mechanical load via sliding of the flexible linker region to avoid excessive stretching of chromatin. When Lamin A/C is deleted, the sensitivity of chromatin to mechanical force increases significantly, and the effect of mechanical force-induced gene activation (such as the MKL1-SRF pathway) is significantly amplified [19,130]. From the perspective of structural distribution, type A and B-type nuclear lamins construct differentiated fibrous network structures [123]. Among them, B-type nuclear lamins are mainly distributed in the perinuclear region, which is closely related to transcriptionally inactive chromatin regions, while Lamin A/C exists in both the perinuclear region and the inner side of the nucleus, covering heterochromatin and euchromatin regions [131,132]. The dynamic activities of the two types of proteins are interactive: the deletion of A-type nuclear lamins will induce the remodeling of the network structure of B-type nuclear lamins, and vice versa, suggesting that they participate in the chromatin remodeling process synergistically [133]. Nuclear lamins regulate the spatial conformation of chromatin and epigenetic status by constructing a perinuclear mechanical support network and mediating chromatin anchoring: the fiber network formed by nuclear lamins provides anchoring sites for chromatin, among which Lamin A/C and Lamin B directly interact with chromatin, enrich heterochromatin in LADs, and maintain the gene silencing state through spatial isolation to limit the binding of transcription factors to target genes [122,134,135]. Furthermore, the interaction between LADs and the nuclear lamina is involved in regulating local chromatin accessibility, and affects the radial arrangement of chromosome territories and the spatial organization of A/B chromatin compartments as an important spatial constraint factor; mechanical force can be transmitted to the nuclear lamina through the LINC complex, reshape the LADs–chromatin interaction and affect the 3D genome folding state [136]. Studies [137] have demonstrated that the spatial rearrangement of chromatin mediated by the nuclear lamina plays a decisive role in cell fate transition: during the differentiation of cardiac progenitor cells, the cardiac gene cluster detaches from the perinuclear region and gains transcriptional activity. This migration process is regulated by the expression level of Lamin A/C and *HDAC3*-dependent heterochromatin anchoring—inhibiting *HDAC3* or down-regulating Lamin A/C expression releases these genes into the transcriptionally active nuclear interior, thereby enhancing differentiation efficiency; on the contrary, forcing gene localization to the perinuclear region blocks the differentiation process. The precise regulation of chromatin epigenetic status by nuclear lamins is the core epigenetic checkpoint for embryonic development and adult tissue lineage differentiation. During myogenic differentiation, Lamin A/C realizes the bidirectional epigenetic regulation of pluripotency-related genes and key genes for myogenic differentiation through the dynamic remodeling of lineage-specific LADs. The results of genome-wide LAD mapping by DNA Adenine Methyltransferase Identification Sequencing (DamID-seq) and epigenetic modification analysis by Chromatin Immunoprecipitation Sequencing (ChIP-seq) show that wild-type Lamin A can anchor pluripotency-related genes such as *Sox2* to the perinuclear heterochromatin region during the differentiation of myoblasts, and achieve transcriptional silencing by gradually establishing H3K9me3 repressive epigenetic modification at the target sites; at the same time, it can make key myogenic genes such as *MYOG* (encoding myogenin) dissociate from LADs, separate from the perinuclear heterochromatin environment and enter the intranuclear euchromatin region, and then gain transcriptional activity [138]. The *LMNA* missense mutations related to Emery–Dreifuss muscular dystrophy (EDMD), taking the experimentally verified p.R453W mutation and the supplementary verified p.H222P mutation as examples, will destroy the above developmental epigenetic conversion program, resulting in the abnormal continuous expression of pluripotency genes and the blockade of the myogenic differentiation program, which is the core epigenetic driving mechanism of the muscular dystrophy phenotype related to *LMNA* mutation [138]. The above epigenetic regulation process can be directly regulated by mechanical microenvironment signals such as extracellular matrix stiffness, which is the core molecular basis for mechanical force to regulate the fate decision of skeletal muscle cells through nuclear lamins. Lamin A/C can realize the epigenetic regulation of cardiomyocyte differentiation through the dynamic remodeling of LADs. During normal development, Lamin A/C anchors non-myocardial lineage genes and cell cycle-related genes to the perinuclear heterochromatin region to maintain their silencing, ensuring the normal differentiation of cardiomyocytes and the exit of terminal cell cycle. Pathogenic mutations of *LMNA* will disrupt this regulation, leading to the dissociation and abnormal activation of target genes, causing cardiomyocyte differentiation disorder and functional abnormality, and finally inducing *LMNA*-related cardiomyopathy and heart failure [139,140]. This mechanism is also a key link for the periodic contractile mechanical stress of myocardial tissue to regulate cardiomyocyte homeostasis through nuclear lamins, and its abnormality will directly lead to the failure of mechanical signal transduction, which is an important pathological basis for the occurrence of related cardiomyopathy. B-type nuclear lamins have irreplaceable specificity of epigenetic regulation in the development of the nervous system. Lamin B1 and Lamin B2 have a clear functional division in chromatin binding. Lamin B1 preferentially binds to facultative heterochromatin, and Lamin B2 specifically anchors constitutive heterochromatin. The genes in their binding regions are enriched in neuronal development pathways, which provides an epigenetic basis for their non-redundant functions related to neural development [54]. The farnesylation modification of Lamin B1 is very important for neuronal migration. Lamin B1 that cannot undergo farnesylation modification will lead to the separation of neuronal nuclear lamina and chromatin, causing abnormal development of the cerebral cortex and perinatal lethality in mice [38]. The heterozygous pathogenic mutations of Lamin B1 and Lamin B2 will lead to abnormal nuclear lamina structure and organization, and imbalance of nuclear morphology homeostasis, which is a new pathogenic mechanism of primary microcephaly, a severe neurodevelopmental disorder, and defines a new laminopathy subtype related to B-type nuclear lamins [141]. In addition, the expression level of Lamin B1 is very important for the development and differentiation of oligodendrocytes. The deletion of the oligodendrocyte-specific silencer upstream of the *LMNB1* gene or the repeat amplification of the whole gene will lead to abnormal overexpression of Lamin B1, cause dysregulation of myelin-related gene expression and impaired oligodendrocyte differentiation, and finally induce Autosomal Dominant Leukodystrophy with Autonomic Disease (ADLD) in adults. Among them, the disorder of chromatin conformation and gene expression mediated by Lamin B1 overexpression is the core basic mechanism of this disease [142,143]. The above neural development-related epigenetic regulation processes are highly dependent on the mechanosensation and transduction functions of B-type nuclear lamins to mechanical force signals, and their functional abnormalities will directly impair the responsiveness to mechanical signals of the microenvironment during neuronal migration and differentiation, and then cause neurodevelopmental disorders.

In response to mechanical signals, the viscoelastic characteristics of the extracellular matrix are transmitted to the nucleus through the integrin–cytoskeleton pathway [62]. This signal will simultaneously regulate the functions of type A/B nuclear lamins to synergistically drive chromatin remodeling. On the one hand, after mechanical force is transmitted to the nuclear lamina through the LINC complex, it can directly induce changes in Lamin B1 and chromatin Topologically Associating Domains (TADs), and drive three-dimensional chromatin remodeling—from A/B compartment conversion caused by the dissociation of perinuclear LADs and changes in TAD boundary insulation to enhanced promoter-enhancer looping inside TADs, which promotes gene transcription [46,135,144]. This process cooperates with the strain buffering function of the LINC complex to ensure the specificity of chromatin remodeling [63]. On the other hand, transcription factors (such as MKL1) released after chromatin decompaction can enhance nuclear stiffness by activating *LMNA* gene expression, forming a positive feedback of “force–nuclear stiffness–epigenetics” [145,146]. At the same time, this transduction pathway will directly induce the down-regulation of Lamin A/C expression accompanied by increased nuclear envelope folds, reduced nuclear stiffness and enhanced deformability, which will lead to a significant decrease in chromatin compaction degree and improved accessibility [122,147,148,149]. This process is accompanied by increased activity of histone acetyltransferases (such as enrichment of H3K27ac markers) and inhibition of deacetylase function, and finally promotes the accumulation of open chromatin epigenetic markers [123]. When Lamin A/C is deleted, the movement rate of chromatin is accelerated and shows disordered characteristics, which can easily cause DNA strand entanglement and damage the order of replication and transcription [24,150]. Normally expressed Lamin A/C promotes the enrichment of repressive epigenetic marks (such as H3K9me3) by anchoring heterochromatin to the perinuclear region, and maintains the structural stability of heterochromatin [151]. Studies [152] have shown that the disintegration of the nuclear lamina structure in senescent cells will lead to the “erosion” of heterochromatin, weaken the interaction efficiency between heterochromatin and the nuclear envelope, and then lead to chromatin relaxation and abnormal gene activation. Ranade et al. [153] found that simultaneous knockdown of Lamin A/C and Emerin will enhance the migration ability of chromatin to the intranuclear region, leading to the mislocalization of chromosome regions and the dysregulation of candidate genes (such as KLK10, BCL2L12 and MADH2), which further reveals the regulatory role of nuclear lamins on genome stability.

In summary, the core mechanism and biological effects of mechanical force transduction mediated by nuclear lamina-related structures are summarized in Table 2.

## 4. Discussion

Nuclear lamins serve as the core molecular bridge linking the extracellular mechanical microenvironment to intranuclear gene regulation, and lamin-mediated mechanosensation and signal transduction are core scientific questions in the field of cellular mechanobiology. Existing studies have thoroughly delineated the cascade regulatory network of mechanical force transduction across the nuclear envelope mediated by the “LINC complex–nuclear lamina–chromatin” axis, and clarified the specific functional division of different subtypes of nuclear lamins in mechanical sensing, signal transmission and effect conversion. A general consensus has been established in existing studies that nuclear lamins are not simply nuclear structural support proteins, but rather core regulatory molecules with the dual functions of “mechanosensor” and “signal transduction hub”. They realize the biological transformation of mechanical signals through two core pathways—A-type nuclear lamins Lamin A/C directly determine the mechanical stiffness of the nucleus and dominate the direct intranuclear transmission of mechanical load [95,98]; B-type nuclear lamins Lamin B1/B2 indirectly regulate the epigenetic remodeling induced by mechanical signals through the non-redundant chromatin anchoring function [54,55]. The two work together to complete the accurate conversion from extracellular physical signals to intranuclear gene expression regulation. Most importantly, the latest finding that Lamin A/C distributed in the nucleoplasm also bears significant mechanical tension has challenged the long-standing view that the mechanical function of nuclear lamins is limited to the nuclear envelope region. It provides a key theoretical basis for redefining the global mechanical response network in the nucleus, and also provides an important new perspective for in-depth analysis of the molecular mechanism of mechanical force rapidly regulating genome-wide transcriptional activity [5].

The structural diversity and functional specificity of nuclear lamin subtypes are the core entry points for analyzing their tissue-specific biological effects and disease phenotypes. Existing studies have confirmed that the functional differentiation of each subtype of nuclear lamins has a clear structural basis and evolutionary conservation. Although Lamin A and Lamin C are splicing products of the same gene, they form spatial differentiation of nuclear envelope anchoring and nucleoplasmic distribution due to differences in post-translational modification, and show non-redundant functions in nuclear structure reconstruction and mechanical sensitivity regulation [23,25]. Lamin B1 and Lamin B2 realize the partition regulation of facultative heterochromatin and constitutive heterochromatin through differentiated histone modification preferences and DNA sequence binding characteristics. This functional division shows irreplaceable specificity in the development of the nervous system [54,55]. The germ cell-specific subtypes adapt to the special mechanical requirements during meiosis and spermatogenesis through the specific reconstruction of the structural domain, which further confirms the core law of “structure determines function” [36,57]. More importantly, the subtype-specific functional division directly explains the tissue-specific phenotype of laminopathy—pathogenic mutations of the *LMNA* gene mainly involve high-stiffness tissues such as myocardium and skeletal muscle that continuously bear mechanical stress, and induce diseases such as dilated cardiomyopathy and Emery–Dreifuss muscular dystrophy [89,138,154], while the functional abnormalities of *LMNB1*/*LMNB2* mainly lead to neurodevelopmental disorders, such as primary microcephaly and leukodystrophy [141,142]. However, there are still key scientific problems that have not been clarified in this field: how differences in the expression profiles of nuclear lamin subtypes across different tissues determine the sensitivity threshold of cells to mechanical microenvironment stimulation; what the compensation and interaction mechanisms of type A and B-type nuclear lamins are in the pathological state, and why they cannot offset the functional defects caused by pathogenic mutations [133]; and how nuclear lamins achieve tissue-specific and precise regulation of chromatin epigenetics in different lineage cells. These questions remain the core directions that need to be investigated in future research [39].

The two-way interaction between mechanical signals and epigenetic regulation is the core molecular mechanism of cell fate determination mediated by nuclear lamins, and also the core research direction in the current field of cellular mechanobiology. Existing studies confirm that nuclear lamins are not passive transmitters of mechanical signals, but rather active participants in the mechanical force-induced three-dimensional genome reconstruction and epigenetic state regulation through direct interactions with chromatin [14,15]. Under physiological conditions, nuclear lamins anchor heterochromatin to the perinuclear region through LADs, and maintain the gene silencing state through spatial isolation [122,134]. After extracellular mechanical force stimulation is transmitted to the nuclear lamina through the LINC complex, it can quickly trigger the dissociation of LADs from the nuclear lamina, the remodeling of chromatin spatial conformation, and the dynamic redistribution of key epigenetic markers such as H3K9me3 and H3K27ac, and then realize the precise regulation of cell differentiation, proliferation, senescence and other life activities [123,136]. This positive feedback loop of “force–nuclear stiffness–epigenetics” plays a decisive role in stem cell lineage differentiation, cardiomyocyte development and aging process. For example, the differentiation fate of mesenchymal stem cells is directly determined by the extracellular matrix stiffness through the epigenetic regulation mediated by Lamin A/C [137,148]. At the same time, the post-translational modifications of nuclear lamins (phosphorylation, farnesylation, etc.) are the key molecular switches connecting mechanical signals and epigenetic regulation. Mechanical force can dynamically change their polymerization ability, nuclear positioning and chromatin binding affinity by regulating the post-translational modification state of nuclear lamins, and then realize the bidirectional regulation of mechanical signal transduction efficiency [8,62,68]. However, there are still obvious gaps in the research in this field: how mechanical force signals realize the precise recruitment and activity regulation of epigenetic modification enzymes through nuclear lamins; whether different types of mechanical stimulation (stretching, compression, fluid shear force) will induce differentiated epigenetic responses through nuclear lamins; and how the crosstalk between post-translational modifications of nuclear lamins synergistically regulates nuclear mechanical homeostasis and epigenetic status. These mechanisms remain to be further elucidated through functional experiments.

Although significant breakthroughs have been made in elucidating the mechanical force transduction mechanism mediated by nuclear lamins, the current research in this field still has many limitations and challenges. At the methodological level, most of the existing studies are based on in vitro two-dimensional cultured cell lines and static mechanical stimulation models, which cannot fully recapitulate the spatiotemporal heterogeneity of periodic mechanical stress, three-dimensional matrix microenvironment and fluid shear force in the in vivo tissue microenvironment. The dynamic mechanical response and signal transduction mechanism of nuclear lamins under in vivo physiological conditions still lack direct verification at the living level [155]. At the level of the universality of research, most of the current studies on the mechanical function of nuclear lamins focus on a few cell types such as cardiomyocytes and mesenchymal stem cells. Systematic research on whether their mechanical transduction mechanism in immune cells, nerve cells and tumor cells is conserved and on the dynamic changes in their functions in different pathological microenvironments is still lacking. At the level of mechanism analysis, the regulatory mechanism of the mechanical load of Lamin A/C in the nucleoplasm, its functional division from the nuclear lamina under the nuclear envelope, and its abnormal changes in the pathological state are still in the initial stage [5]; how the mechanical coupling efficiency between the LINC complex–nuclear lamina–chromatin is synergistically regulated by the chromatin compaction state and the interaction of nuclear envelope-related proteins and its detailed molecular mechanism still need to be further clarified [10,11]. Notably, the rapid development of emerging technologies such as nanobody-mediated FRET tension sensing, in vivo nuclear mechanics imaging, single-cell multi-omics and super-resolution imaging in recent years provides technical support for breaking through the above research limitations [5,156,157]. In the future, integrating multidisciplinary technical means to analyze the mechanical regulation mechanism of nuclear lamins in a model closer to the physiological state will be an important development direction in this field.

Abnormalities of the mechanical force transduction pathway mediated by nuclear lamins are the core pathogenic mechanism of many diseases, and also provide a new targeted direction for the treatment of related diseases. At present, a number of preclinical studies have confirmed the therapeutic potential of targeting this pathway: farnesyltransferase inhibitors can improve the nuclear structure defects and mechanical homeostasis imbalance caused by abnormal accumulation of progerin in progeria models [32]; inhibition of *miR-124-3p* can reverse the apoptosis of vascular smooth muscle cells induced by mechanical stretching, providing an intervention target for vascular mechanical injury-related diseases [158]; and *SIRT1* overexpression can alleviate myocardial dysfunction caused by Lamin A/C deletion, providing a new strategy for the treatment of *LMNA*-related cardiomyopathy [124]. However, the clinical translation of this pathway still faces three core challenges: first, nuclear lamins are widely expressed in various tissues of the whole body. How to achieve tissue-specific delivery of therapeutic targets and avoid off-target effects caused by interfering with normal physiological functions is the primary challenge for clinical translation [27]. Second, the nuclear lamina mechanical regulation pathway has extensive crosstalk with multiple intracellular signaling pathways. Determining how to accurately regulate the abnormal pathways related to pathology without affecting their basic physiological functions of maintaining nuclear structure and genome stability still needs more in-depth mechanism analysis. Third, most of the existing research results are based on rodent animal models, and there are significant species differences between rodents and humans in the expression regulation of nuclear lamins and the mechanical microenvironment of tissues. The effectiveness and safety of the research results translated to clinical practice still need to be fully verified. In the future, the development of tissue-specific targeted delivery systems, screening of subtype-specific functional regulators of nuclear lamins, and carrying out pharmacodynamic verification based on human cells and organoid models are expected to advance the clinical translation and application of the nuclear lamina mechanical regulatory mechanism.

In summary, the existing studies on the molecular characteristics and subtype functional division of nuclear lamins have completely constructed the cascade regulatory network of “LINC complex–nuclear lamina–chromatin”, and clarified their core role in the transnuclear envelope transduction of mechanical force, chromatin remodeling and epigenetic regulation. As the molecular bridge between mechanosensation and signal transduction, nuclear lamins are critical for maintaining normal cellular physiological activities via their functional homeostasis, and their dysregulation is a core driver of the onset and progression of numerous human diseases [4,125]. Future studies should further focus on the tissue-specific regulatory mechanism of nuclear lamin subtypes, the crosstalk network between mechanical signals and epigenetics, and the feasibility of clinical translation targeting this pathway, so as to provide a more solid theoretical basis and experimental basis for the precise prevention and treatment of laminopathy, cardiovascular diseases and other related diseases.

## Figures and Tables

**Table 1:** Structural characteristics and mechanical functional division of each subtype of nuclear lamins.

Subtype Category	Isoform	Encoding Gene	Core Structural Differences	Core Mechanical Functions and Biological Effects
A-type nuclear lamins (core major subtypes)	Lamin A	*LMNA*	Contains a complete α-helical rod domain and a C-terminal CaaX motif, requiring post-translational modifications such as farnesylation for maturation	1. Directly determines the mechanical stiffness of the nucleus, showing a linear positive correlation with the nuclear bulk modulus; 2. Anchors the inner nuclear envelope and maintains the tensile stability of nuclear structure; 3. Specifically binds to SUN1 and mediates the transnuclear envelope mechanical transmission of the LINC complex; 4. Regulates chromatin anchoring and epigenetic status
	Lamin C	*LMNA*	An alternative splicing product of *LMNA*, without a CaaX motif, no farnesylation modification required	1. Forms heterodimers with Lamin A to enhance the mechanical sensitivity of the filament network; 2. Dominates nuclear structure reconstruction and chromatin spatial organization after cell mitosis; 3. Has the strongest linear correlation with cellular mechanical phenotype, and regulates nuclear deformability
B-type nuclear lamins (core major subtypes)	Lamin B1	*LMNB1*	Contains a C-terminal CaaX motif, requires farnesylation and carboxymethylation for maturation, forms a filament network with larger grid side length	1. Preferentially anchors facultative heterochromatin modified by H3K27me3 and maintains chromatin compaction state and perinuclear anchoring; 2. Indirectly maintains the mechanical homeostasis of nuclear structure and buffers mechanical stress; 3. Regulates neuronal migration and oligodendrocyte differentiation, and its abnormal expression causes leukodystrophy
	Lamin B2	*LMNB2*	Contains a C-terminal CaaX motif, the post-translational modification is consistent with B1, forms a filament network with larger gaps and irregular structure	1. Preferentially anchors facultative heterochromatin modified by H3K9me3 to maintain genome stability; 2. Ensures the accuracy of chromosome segregation in mitosis, and maintains the structural integrity of the nuclear envelope and nucleolus; 3. Its dysfunction is closely related to neurodevelopmental disorders such as primary microcephaly
Special/variant/germ cell-specific subtypes	Lamin Aδ10	*LMNA*	Deletion of exon 10, shortened α-helical rod domain	Increases flexibility of the filament network and significantly reduces mechanical load conduction efficiency, but the specific physiological function is still unclear
	Lamin Aδ50 (Progerin)	*LMNA*	Deletion of the terminal 150 amino acids, retains the farnesylation site, compact tail domain	Anchors the inner nuclear envelope and can buffer local mechanical tension, but its excessive accumulation disrupts the uniformity of the nuclear lamina network and weakens the overall mechanical stability of the nucleus, which is the core pathogenic factor of Hutchinson–Gilford progeria syndrome
	Lamin C2	*LMNA*	Deletion of 112 amino acids at the N-terminal	Mediates telomere–chromosome mechanical transmission, forms specific domains in the nuclear envelope, and supports meiotic homologous recombination
	Lamin B3	*LMNB2*	Shortened α-helical rod domain, N-terminal sequence replaced by specific amino acids	Reduces the structural stiffness of the perinuclear region, and provides mechanical flexibility for nuclear recombination during spermatogenesis

**Table 2:** Mechanism and biological effects of core components of nuclear lamina-related mechanical force transduction.

Core Pathway Link	Key Components	Specific Molecular Mechanism Mediated by Mechanical Force	Final Biological Effects
Transnuclear envelope mechanical transmission link	LINC complex (SUN proteins of INM, KASH proteins of ONM)	1. As the core molecular bridge for the transnuclear envelope transmission of mechanical force, it accurately transmits the extracellular mechanical signals sensed by the cytoskeleton to the nuclear lamina through the interaction of SUN-KASH proteins; 2. It is not a rigid structure, and can buffer mechanical overload through disulfide bond rearrangement and conformational rearrangement to maintain the stability of nuclear envelope structure; 3. Different subtypes have specificity: SUN1 preferentially binds to Lamin A specifically, while SUN2 regulates actin homeostasis; and Nesprin-1/2 bridges F-actin, Nesprin-3 connects intermediate filaments, and Nesprin-4 binds to microtubules, realizing the differential transmission of different types of mechanical force	1. Realize efficient and specific transmission of mechanical signals between cytoplasm and nucleus, and lay a structural foundation for intranuclear chromatin remodeling and gene expression regulation; 2. Maintain nuclear positioning and cell polarity, and regulate cell migration and differentiation processes; 3. The abnormal coupling between it and the nuclear lamina is an important pathogenic mechanism of laminopathy and cardiomyopathy
Core hub of mechanical sensing and transduction	Nuclear lamins (type A Lamin A/C, type B Lamin B1/B2 and specific subtypes)	1. Have the dual functions of “mechanosensor” and “signal transduction hub”, and form a two-way feedback between nuclear stiffness and mechanical force sensing: mechanical force can regulate the polymerization state of nuclear lamins through phosphorylation modification, and reversely adjust nuclear stiffness; 2. Type A Lamin A/C: directly determines the mechanical stiffness of the nucleus, and is the core carrier of intranuclear mechanical signal transmission. Lamin A/C in the nucleoplasm also bears significant mechanical tension; 3. Type B Lamin B1/B2: indirectly maintains the mechanical homeostasis of nuclear structure through perinuclear anchoring of chromatin, and regulates chromatin remodeling induced by mechanical force; 4. Interact with nuclear envelope proteins such as Emerin, BAF and LAP2α to build a complete mechanical force transmission network	1. Physiological level: ensure the structural integrity of the nucleus under mechanical stimulation, avoid genome disturbance, regulate gene expression through chromatin anchoring, and determine core life activities such as stem cell differentiation, cell cycle and senescence; 2. Pathological level: coding gene mutation, abnormal expression or disorder of post-translational modification will directly lead to the failure of mechanical transduction, causing a variety of laminopathies such as progeria, dilated cardiomyopathy, muscular dystrophy, and neurodevelopmental disorders
Mechanical signal effect conversion link	Chromatin remodeling and epigenetic regulation system	1. After mechanical force is transmitted through nuclear lamins, it directly induces chromatin stretching and the dissociation of LADs from the nuclear lamina, changing the spatial conformation of chromatin and transcriptional accessibility; 2. Regulate the intranuclear localization and activity of histone modifying enzymes, and dynamically change the genome distribution of epigenetic markers such as H3K9me3, H3K27ac and H4K16ac; 3. Mediate the conversion of chromatin A/B compartments and the remodeling of TAD boundaries, and realize the accurate conversion of mechanical signals to transcriptional regulation	1. Quickly convert physical mechanical signals into biological effects of gene expression, and determine the direction of cell fate; 2. Maintain the stability of higher-order chromatin structure and genome integrity; 3. Abnormalities in this regulatory link will lead to cell differentiation disorders, accelerated senescence, and abnormal activation of disease-related genes, which is the core molecular basis of pathological changes caused by abnormal mechanical microenvironments
Auxiliary regulatory link	Nuclear envelope-related proteins (Emerin, BAF, LAP2α/β)	1. Closely interact with nuclear lamins, assist the nuclear lamina in receiving and transmitting mechanical signals, and enhance the efficiency of mechanical transduction; 2. BAF stabilizes the nuclear envelope-chromatin connection by bridging DNA and nuclear lamins; LAP2α maintains the dynamic balance of Lamin A/C in the nucleoplasm, and regulates the balance of nuclear flexibility and rigidity; and LAP2β mediates the transmission of mechanical force from the nuclear lamina to chromatin	1. Synergistically maintain the integrity of the nuclear envelope and nuclear mechanical homeostasis; 2. Ensure the efficient transmission of mechanical force signals to chromatin; 3. Their dysfunction will aggravate the pathological phenotype caused by nuclear lamin mutations

## Data Availability

All data generated or analyzed during this study are included in this published article.
